# Exploring Machine Learning Algorithms to Unveil Genomic Regions Associated With Resistance to Southern Root-Knot Nematode in Soybeans

**DOI:** 10.3389/fpls.2022.883280

**Published:** 2022-05-03

**Authors:** Caio Canella Vieira, Jing Zhou, Mariola Usovsky, Tri Vuong, Amanda D. Howland, Dongho Lee, Zenglu Li, Jianfeng Zhou, Grover Shannon, Henry T. Nguyen, Pengyin Chen

**Affiliations:** ^1^Fisher Delta Research, Extension, and Education Center, Division of Plant Science and Technology, University of Missouri, Portageville, MO, United States; ^2^Biological Systems Engineering, University of Wisconsin–Madison, Madison, WI, United States; ^3^Division of Plant Science and Technology, University of Missouri, Columbia, MO, United States; ^4^Department of Entomology, College of Agriculture and Natural Resources, Michigan State University, East Lansing, MI, United States; ^5^Institute of Plant Breeding, Genetics, and Genomics, College of Agricultural and Environmental Sciences, University of Georgia, Athens, GA, United States

**Keywords:** machine learning, feature selection, GWAS, soybean, root-knot nematode

## Abstract

Southern root-knot nematode [SRKN, *Meloidogyne incognita* (Kofold & White) Chitwood] is a plant-parasitic nematode challenging to control due to its short life cycle, a wide range of hosts, and limited management options, of which genetic resistance is the main option to efficiently control the damage caused by SRKN. To date, a major quantitative trait locus (QTL) mapped on chromosome (Chr.) 10 plays an essential role in resistance to SRKN in soybean varieties. The confidence of discovered trait-loci associations by traditional methods is often limited by the assumptions of individual single nucleotide polymorphisms (SNPs) always acting independently as well as the phenotype following a Gaussian distribution. Therefore, the objective of this study was to conduct machine learning (ML)-based genome-wide association studies (GWAS) utilizing Random Forest (RF) and Support Vector Machine (SVM) algorithms to unveil novel regions of the soybean genome associated with resistance to SRKN. A total of 717 breeding lines derived from 330 unique bi-parental populations were genotyped with the Illumina Infinium BARCSoySNP6K BeadChip and phenotyped for SRKN resistance in a greenhouse. A GWAS pipeline involving a supervised feature dimension reduction based on Variable Importance in Projection (VIP) and SNP detection based on classification accuracy was proposed. Minor effect SNPs were detected by the proposed ML-GWAS methodology but not identified using Bayesian-information and linkage-disequilibrium Iteratively Nested Keyway (BLINK), Fixed and Random Model Circulating Probability Unification (FarmCPU), and Enriched Compressed Mixed Linear Model (ECMLM) models. Besides the genomic region on Chr. 10 that can explain most of SRKN resistance variance, additional minor effects SNPs were also identified on Chrs. 10 and 11. The findings in this study demonstrated that overfitting in GWAS may lead to lower prediction accuracy, and the detection of significant SNPs based on classification accuracy limited false-positive associations. The expansion of the basis of the genetic resistance to SRKN can potentially reduce the selection pressure over the major QTL on Chr. 10 and achieve higher levels of resistance.

## Introduction

Soybean [*Glycine max* (L.) Merr.] represents one of the most essential crops to the world’s economy and food security due to its unique seed composition. As a versatile crop with unprecedented seed composition, soybean is extensively used in the food, feed, and many other industries exploring oil and protein-based products (Vieira and Chen, 2021). Over the last decade, soybean production has increased approximately 40% expanding from 257.8 to 362.1 million metric tons (2010–2020) (USDA United States Department of Agriculture, 2010, 2020). Yearly, this represents an increment of 26.5 kg ha^–1^ in yield (Koester et al., 2014), which can be attributed to genetic improvements as well as advancements in farming technology and management practices (Specht et al., 1999; De Bruin and Pedersen, 2008; Rowntree et al., 2013; Koester et al., 2014). However, many biotic and abiotic stressors can limit soybean yield potential in the United States and around the world.

In the United States, the average annual yield losses caused by soybean diseases are estimated to be over 11% (Hartman et al., 2015), which translates into an average economic loss of approximately $60.66 per acre (Allen et al., 2017). With over 4,100 species of plant-parasitic nematodes around the world (Decraemer and Hunt, 2006), these small parasites are responsible for annual agricultural losses of approximately $160 billion, severely impacting global food security (Abad et al., 2008). Root-knot nematodes (*Meloidogyne* spp.) are considered the most economically important and widely distributed species of plant-parasitic nematode, of which southern root-knot nematode [SRKN, *Meloidogyne incognita* (Kofold & White) Chitwood] has the most scientific and economic importance (Jones et al., 2013). In soybeans, observed symptoms of SRKN are similar to abiotic stressors, including stunted growth, wilting, leaf discoloration, and deformation of the roots. The magnitude of crop losses depends on historical crop rotation and field usage, environmental parameters, initial nematode population density, soil type, and genetic background (Vieira et al., 2021). SRKN is challenging to control due to its short life cycle and high reproductive rates (Trudgill and Blok, 2001). Crop rotation is especially challenging and limited since most flowering plants are hosts to SRKN (Walker, 1995; Trudgill and Blok, 2001; Luc et al., 2005). Chemical approaches used to be an effective management option to control these nematodes, however, most commercial nematicides and soil fumigants were banned due to toxicity to humans, animals, and environments (Abad et al., 2008). Therefore, the use of genetic resistance becomes the most sustainable – economically, environmentally, and socially – alternative to efficiently control the damage caused by SRKN in soybeans (Vieira et al., 2021).

The first genetic mapping of resistance to SRKN in soybean identified two resistance quantitative trait locus (QTL) on chromosomes (Chrs.) 10 and 18 in plant introduction (PI) 96354 (Tamulonis et al., 1997). The combination of these resistance QTLs in PI 96354 was reported to enhance the levels of resistance to SRKN (Li et al., 2001). Additional marker-trait associations have been identified on Chrs. 6 in a soybean variety derived from PI 96354 (Shearin et al., 2009), 7 in soybean variety LS5995 (Fourie et al., 2008), 8 in PI 438489B (Xu et al., 2013), 10 in “Palmetto,” LS5995, PI 96354, PI 438489B, PI 567516C, and PI 567305 (Ha et al., 2004; Fourie et al., 2008; Pham et al., 2013; Xu et al., 2013; Passianotto et al., 2017; Vuong et al., 2021), 13 in PI 438489B, PI 567516C, and PI 567305 (Xu et al., 2013; Jiao et al., 2015; Vuong et al., 2021), 17 in PI 567516C (Jiao et al., 2015), and 18 in PI 96354 (Pham et al., 2013). The effect of combining these marker-trait associations has not been investigated to date. Attempts to analyze gene expression patterns after infection as well as fine-map the genomic region of the major QTL on Chr. 10 identified candidate genes with cell wall modification-related functions including extensin and pectinesterase encoding functions, carbon and energy metabolism, defense-related, transcription factors and proteins encoding, and cell division-related genes (Ibrahim et al., 2011; Eugênia et al., 2012; Beneventi et al., 2013; Pham et al., 2013; Xu et al., 2013; Passianotto et al., 2017). Most genetic mapping studies for SRKN resistance are based on the development of galls in the root system (galling response) of soybean lines reported as categorical variables, often using bi-parental populations with limited molecular marker density and coverage.

Traditional genome-wide association studies (GWAS) identify genomic regions associated with a trait or phenotype of interest from a large group of single nucleotide polymorphisms (SNPs) by linear or logistic regression analysis which is performed separately for each SNP. The resulting *p*-values are then used to rank the SNPs and to select those with a *p*-value smaller than a pre-set significance level threshold (e.g., *p*-value < 0.05 or LOD score of 3.0) (Szymczak et al., 2016). The confidence of discovered trait-loci associations by the traditional methods is often limited by the assumptions of individual SNPs always acting independently, false-positive SNPs identified by linkage disequilibrium, as well as the phenotype following a Gaussian distribution (Korte and Farlow, 2013; Nicholls et al., 2020). Although statistical methodologies to account for epistatic interaction, as well as population relatedness-false associations have been developed (Marchini et al., 2005; Cordell, 2009; Kam-Thong et al., 2011; Liu et al., 2016; Huang et al., 2019), linear model-based genome-wide studies still experience drawbacks from the extensive number of pair-wise tests that need to be performed (Korte and Farlow, 2013). Recently developed machine learning (ML) based GWAS has provided a promising alternative to classical, model-based statistical methods for the selection of important SNPs in datasets where the number of independent variables is far higher than the number of samples that are often seen in genomic studies (Nicholls et al., 2020). ML-based GWAS has the advantage of taking into account the interaction effects between markers, whereas conventional GWAS methodologies are appropriate for detecting markers with large effects on complex traits and underpowered for the simultaneous consideration of a wide range of interconnected biological and physiological processes and mechanisms that constitute the phenotype of interest.

Popular ML models, such as Random Forest (RF) and Support Vector Machine (SVM) have been involved in GWAS for feature (SNPs) selection (Merelli et al., 2013; Szymczak et al., 2016), performance assessment (Vitsios and Petrovski, 2019) and result prioritization (Ning et al., 2015). Though advanced rapidly, ML-based GWAS faces challenges, including high computational expenses and difficulty to interpret and handle the high dimensionality in predictors. Besides, the applications of ML-based GWAS need to be consistently validated with significant associations that make both biological and statistical sense (Nicholls et al., 2020). To the best of our knowledge, ML-based GWAS has been applied in soybean to identify significant marker-trait associations using SVM (Yoosefzadeh-Najafabadi et al., 2021a,b), RF (Zhou et al., 2019; Xavier and Rainey, 2020; Yoosefzadeh-Najafabadi et al., 2021b), and Deep Convolutional Neural Network (CNN) (Liu et al., 2019), of which none was applied on soybean resistance to SRKN. Therefore, the objective of this study was to conduct ML-GWAS utilizing 717 diverse breeding lines derived from 330 unique bi-parental populations with two different algorithms (SVM and RF) to unveil novel regions of the soybean genome regulating the resistance to SRKN (reported as the development of galls in the roots) and contribute to developing enhanced and more durable SRKN resistance.

## Materials and Methods

### Plant Materials and Data Collection

#### Soybean Breeding Lines Panel and Genotyping

A total of 717 breeding lines derived from 330 unique bi-parental populations and developed by the University of Missouri – Fisher Delta Research Center (MU-FDRC) soybean breeding program was used in this study. The MU-FDRC soybean breeding program has historically advanced the field of nematode resistance in soybeans and developed and released multiple soybean lines with enhanced levels of SRKN resistance by combining multiple sources of resistance (Shannon et al., 2019; Chen P. et al., 2021). The lines comprised 5 years (2017–2021) of internal advanced yield trials at the MU-FDRC. Five seeds of each line were grown in a greenhouse, and genomic DNA was extracted from lyophilized young trifoliate leaf tissue (V3) (Fehr et al., 1971) using the Qiagen DNeasy Plant 96 kit (QIAGEN, Valencia, CA, United States) and respective protocol. DNA concentration was quantified with a spectrophotometer (NanoDrop Technologies Inc., Centerville, DE, United States) and normalized at 50 ng/μl. DNA samples were genotyped in the USDA-ARS Soybean Genomics and Improvement Laboratory using the Illumina Infinium BARCSoySNP6K BeadChip (Song et al., 2020). The SNP alleles were called using the Illumina Genome Studio Genotyping Module (Illumina, Inc., San Diego, CA, United States). SNPs were converted to numerical format (0, 1, and 2 for the homozygous minor allele, heterozygous, and homozygous major allele, respectively), and were excluded based on minor allele frequency (MAF) < 0.05 resulting in 4,974 SNPs. The across-genome SNP density was 249, ranging from 191 (Chr. 17) to 327 (Chr. 08).

#### Phenotypic Characterization

Breeding lines were phenotyped for the development of galls in the root system (galling response) in a greenhouse of the University of Georgia from 2017 to 2021 using a well-established protocol as previously described (Hussey and Boerma, 1981). The resistant and susceptible standard checks “Bossier,” “CNS” (PI 548445), “GaSoy17” (PI 553046), G93-9009 (Luzzi et al., 1996), and “Haskell” (PI 572238) were included in the bioassays. Three seeds of each line were planted in four replications in Ray Leach Cone-tainers (20.6 cm long cones) and filled with fumigated sandy loam soil. Plants were thinned to one seedling per cone-tainer after emergence and then inoculated with 3,000 SRKN eggs (race 3) after 10 days. Forty days after inoculation, the plants were uprooted. The roots were washed free of soil, and the galls were counted (Hussey and Boerma, 1981). The number of galls on the resistant and susceptible standard checks were used to determine rating scales for these lines, where 1 < 10 galls per plant, 2 = 11 to 20, 3 = 21 to 30, 4 = 31 to 40, and 5 >40 galls. For classification purposes, lines were considered tolerant when <20 galls per plant, moderate >20, <40, and susceptible >40 galls per plant.

### Genome-Wide Association Study

#### Single Nucleotide Polymorphism Feature Selection

To select SNPs that were significantly associated with SRKN resistance, a Partial Least Square (PLS) (Wold, 1966) model was fitted using the 4,974 SNPs as predictors and the number of galls in the root as responses. PLS models have the advantage to reduce the variability and instability of estimated responses caused by multicollinearity among predictors (Zhou et al., 2019; James et al., 2021). Additionally, PLS creates linear combinations (known as components) of the original predictor variables (the SNPs) to explain the observed variability in the responses (the galling response). Coefficients associated with the components were trained with 10-fold cross-validation to reach a minimum validation error. The relative importance of these variables in the components was retrieved by calling the Variable Importance in Projection (VIP) scores in the PLR model fitting results. The PLS model fitting was conducted in *R* (R Core Team, 2021) using “*plsregress*” function in the “*pls*” package (Mevik and Wehrens, 2007) and the VIP scores were returned by calling the “*VIP*” function in the “*plsVarSel*” package (Mehmood et al., 2012).

The SNPs with high VIP scores (>2.0) were kept to be included in the ML-based GWAS and sorted descendingly based on the VIP scores. Starting from the top of the selected SNP list, the Pearson correlations (*r*) of one SNP with the others were calculated, and those with high correlations (| *r*| > 0.5) were removed from the list. The list was updated immediately and the correlations between the following SNP and the others were calculated. The loop ended when the last SNP correlations were calculated.

#### Machine Learning Algorithms

The SNPs with high VIP values and low correlations with other SNPs were further selected by ML models in a forward stepwise selection loop. The selection loop started from taking single SNPs as model predictors and the development of galls in the root system as responses. Each of the models was evaluated with 5-folder cross-validation and their classification accuracy was recorded. The overall accuracy of each model was calculated using Eq. 1. Class accuracy, which represents the ratio of correctly predicted instances and all the instances, was calculated using Eq. 2. Precision, which indicates the proportion of predicted presences, was calculated using Eq. 3, and specificity, which indicates the ratio of correctly predicted negative classes was calculated using Eq. 4. Matthews Correlation Coefficient (MCC) was calculated using Eq. 5. The SNP with the highest accuracy in the previous loop was kept in the later loop and evaluated with an additional SNP from the list of significant SNPs. The loop ended when no gain in the classification accuracy was observed and output the best combination of SNPs. To assess the effect of potential overfitting on the predictive accuracy of both SVM and RF models, the loop was extended to all selected predictors and accuracy metrics were calculated for each model.


(1) $$\begin{array}{c} OverallAccuracy=\\ \frac{No.ofsamplesclassifiedcorrectlyinatestset}{TotalNo.ofsamplesinatestset}\times 100\% \end{array}$$

(2) $$ClassAccuracy=\frac{TP+TN}{TP+TN+FP+FN}$$

(3) $$Precision=\frac{TP}{TP+FP}$$

(4) $$Specificity=\frac{TN}{TN+FP}$$

(5) $$\begin{array}{c} MCC=\\ \frac{\left(TP\times TN\right)-\left(FP\times FN\right)}{\sqrt{\left(TP+FP\right)\left(TP+FN\right)\left(TN+FP\right)\left(TN+FN\right)}} \end{array}$$

where, TP, True Positive; TN, True Negative; FP, False Positive; FN, False Negative.

Two models were used for the multi-class problem, namely SVM and RF. The two models were selected due to their high effectiveness in high dimensional cases where the number of predictors is greater than the number of samples, as well as a good balance between the variance-bias trade-off (James et al., 2021). RF is a tree-based supervised learning algorithm based on assembling multiple decision trees. It can perform feature selection and generate uncorrelated decision trees by randomly dropping a set of input variables so that it allows to model a high number of features in the data (Breiman, 2001). The SVM model works well in classification problems by placing flexible hyperplanes among classes. The model offers controllability to users by combinations of tunable parameters to ensure model performance and avoid potential overfitting.

The RF model was called by the “*randomForest*” function in the “*randomForest*” package (Liaw and Wiener, 2002) with sqrt(p) (where p is the number of variables) variables randomly sampled as candidates at each split. The SVM model was fitted by the “*svm*” function in the “*e1071*” (Meyer et al., 2021) package and the kernel was defined as “radial.” The best combination of trainable parameters in SVM (i.e., gamma and cost) were returned automatically by calling the “*tune*” function. The model was turned by going through a grid search for cost (the margin softness parameter) from 0.01, 0.1, 1, 10, 100, and 1,000 and gamma (the variance-bias tradeoff parameter) from 0.0001, 0.001, 0.01, 0.5, and 1. In addition, to compare the efficacy of the proposed methodology in detecting significant SNPs, GWAS was conducted using the package GAPIT (Lipka et al., 2012) with the models Enriched Compressed Mixed Linear Model (ECMLM) (Li et al., 2014), Fixed and Random Model Circulating Probability Unification (FarmCPU) (Liu et al., 2016), and Bayesian-information and linkage-disequilibrium Iteratively Nested Keyway (BLINK) (Huang et al., 2019). The threshold of significance was calculated based on the false discovery rate (FDR)-adjusted *p*-values to reduce false-positive associations (Benjamini and Hochberg, 1995).

Compressed Mixed Linear Model (CMLM) groups individuals based on kinship replacing the genetic effects of individuals in the regular mixed linear model (MLM) with the genetic effects of the corresponding groups. In ECMLM, additional algorithms are provided to cluster individuals into groups including the average and Ward methods. The detailed methodology can be found in Li et al. (2014). FarmCPU was developed to eliminate the confounding effect between kinship in an MLM and genes underlying a trait of interest by substituting the kinship with a set of markers associated with the causal genes. The set of the associated markers is fitted as a fixed effect in a fixed-effect model for testing markers one at a time across the genome. This set is optimized in a maximum likelihood method in an MLM with variance and covariance structure defined by the associated markers to minimize the risk of overfitting. Liu et al. (2016) described the methodology in detail. BLINK is a methodology based on FarmCPU targeting the major limitations of the latter. BLINK does not assume that causal genes are evenly distributed across the genome by directly working on markers instead of bins. Markers that are in linkage disequilibrium (LD) with the most significant marker are excluded until no marker can be excluded. In addition, BLINK uses Bayesian Information Content (BIC) of a fixed-effect model to approximate the maximum likelihood of a random effect model to select the associated markers among the ones that remained after the exclusion based on LD. The detailed methodology can be found in Huang et al. (2019).

## Results

### Phenotypic Distribution and Feature Selection

**FIGURE 1:**
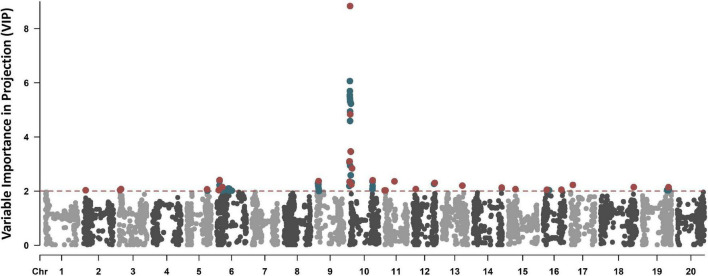
Variable Importance in Projection (VIP)-based Manhattan plot of the 4,974 SNPs. The SNPs with VIP scores higher than 2.0 are highlighted in blue, and the 29 non-correlated SNPs with VIP scores higher than 2.0 selected to be used in the ML-based GWAS are colored in red.

A total of 186 genotypes were scored as resistant to SRKN (average score of 1.3), 105 as moderate (average score of 3.0), and 426 as susceptible (average score of 4.9). The distribution was unbalanced as the susceptible (59.4%) lines largely outnumbered the resistant (25.9%) and moderate (14.6%) lines. The average VIP scores across the 4,974 SNPs was 0.89, of which 2,167 SNPs showed VIP scores above the standard threshold of 1.0 (Figure 1). The PLS-VIP method is often used when multicollinearity is present among features (Chong and Jun, 2005), which is a common scenario with high-density SNP datasets. The method ranks the features based on their importance toward the aggregate index (*D*_*e*_). Since the average of squared VIP scores equals one, a score greater than 1.0 is generally used as a threshold for selecting features that contribute the most toward the aggregate index (Chong and Jun, 2005; Cocchi et al., 2018). Alternative values include increasing the threshold to 2.0–3.0 or adjusting based on the average of VIP values (Cocchi et al., 2018). In this study, we used the threshold of 2.0 considering the high multicollinearity between SNPs, as well as the relatively high average VIP scores in this dataset. To reduce model overfitting and correlated features, SNPs with pair-wise Pearson correlation (| *r|*) higher than 0.5 were eliminated, maintaining the SNP with higher VIP scores. A total of 29 non-correlated SNPs with VIPs higher than 2.0 (range 2.0–8.8) were identified across Chrs. 2, 3, 5, 6, 9, 10, 11, 12, 13, 14, 15, 16, 17, 18, and 19, and selected to be included in the analysis (Figure 1).

### Genome-Wide Association Study Results

#### Machine Learning Genome-Wide Association Studies

The SVM model achieved the highest overall prediction accuracy (0.78) using five SNPs as predictors, including *Gm10-1232205*, *Gm10-2240113*, *Gm10-214458*, *Gm10-1586434*, and *Gm11-63293*. *Gm10-1232205* was the SNP with the highest VIP score (8.83) and yielded a classification accuracy of 0.74 when used as the only predictor. The addition of *Gm10-2240113*, *Gm10-214458*, *Gm10-1586434*, and *Gm11-63293* to *Gm10-1232205* improved the model’s ability to classify resistant, moderate, and susceptible genotypes, with an overall increment in prediction accuracy of 5%. A substantial gain in accuracy was observed in the moderate class, increasing from 0.50 to 0.59 (18%). The precision, which measures the ability of the model to classify a true positive prediction based on the total number of positive predictions, increased for all classes with the addition of the four SNPs, however, a drastic increase in precision was observed in the moderate class (0.00–0.63). In addition, specificity, which represents the proportion of true negative predictions by the total number of negative predictions, increased proportionally for the resistant and susceptible classes (7.5 and 6.8%, respectively). Interestingly, a substantial decrease in overall prediction accuracy was observed with the further addition of predictors, which can be attributed to the overfitting of the training set and consequently poor reproducibility in the testing set (Table 1).

**TABLE 1:** Summary of SVM classification accuracy metrics based on the number of predictors.

# SNPs^1^	Overall accuracy^2^	MCC^3^	Resistant			Moderate			Susceptible		
			Accuracy^4^	Precision^5^	Specificity^6^	Accuracy	Precision	Specificity	Accuracy	Precision	Specificity
1	0.74	0.53	0.87	0.55	0.80	0.50	0.00	1.00	0.80	0.84	0.73
2	0.74	0.51	0.85	0.59	0.84	0.52	0.29	0.96	0.80	0.84	0.73
3	0.75	0.54	0.85	0.59	0.84	0.53	0.50	0.98	0.80	0.83	0.71
4	0.76	0.56	0.86	0.62	0.86	0.53	0.50	0.98	0.82	0.84	0.71
**5**	**0.78**	**0.60**	**0.86**	**0.62**	**0.86**	**0.59**	**0.63**	**0.97**	**0.85**	**0.87**	**0.78**
6	0.78	0.59	0.86	0.62	0.86	0.56	0.75	0.99	0.83	0.85	0.73
7	0.77	0.57	0.86	0.62	0.86	0.54	0.67	0.99	0.82	0.84	0.71
8	0.77	0.57	0.86	0.62	0.86	0.54	0.67	0.99	0.82	0.84	0.71
9	0.76	0.56	0.86	0.62	0.86	0.53	0.50	0.98	0.82	0.84	0.71
10	0.76	0.56	0.86	0.60	0.85	0.53	0.50	0.98	0.82	0.85	0.73
11	0.76	0.55	0.86	0.60	0.85	0.52	0.33	0.97	0.83	0.85	0.75
12	0.74	0.52	0.86	0.62	0.86	0.51	0.25	0.95	0.81	0.84	0.73
13	0.76	0.57	0.88	0.63	0.86	0.54	0.38	0.96	0.83	0.86	0.76
14	0.75	0.54	0.86	0.62	0.86	0.53	0.33	0.95	0.82	0.85	0.75
15	0.75	0.54	0.86	0.62	0.86	0.53	0.33	0.95	0.82	0.85	0.75
16	0.75	0.54	0.86	0.62	0.86	0.53	0.33	0.95	0.82	0.85	0.75
17	0.75	0.54	0.86	0.60	0.85	0.52	0.29	0.96	0.82	0.85	0.75
18	0.76	0.55	0.86	0.60	0.85	0.54	0.38	0.96	0.83	0.86	0.76
19	0.76	0.55	0.86	0.60	0.85	0.54	0.38	0.96	0.83	0.86	0.76
20	0.76	0.55	0.86	0.60	0.85	0.54	0.38	0.96	0.83	0.86	0.76
21	0.75	0.54	0.85	0.61	0.86	0.53	0.33	0.95	0.82	0.85	0.75
22	0.74	0.53	0.85	0.57	0.82	0.51	0.25	0.97	0.82	0.85	0.75
23	0.74	0.53	0.86	0.60	0.85	0.51	0.25	0.95	0.82	0.85	0.75
24	0.74	0.53	0.83	0.56	0.82	0.51	0.33	0.98	0.82	0.84	0.73
25	0.74	0.51	0.80	0.64	0.89	0.58	0.38	0.92	0.81	0.84	0.73
26	0.75	0.53	0.82	0.67	0.90	0.60	0.41	0.92	0.81	0.84	0.73
27	0.74	0.53	0.83	0.56	0.82	0.54	0.67	0.99	0.80	0.83	0.71
28	0.74	0.52	0.83	0.57	0.83	0.53	0.50	0.98	0.80	0.83	0.71
29	0.73	0.50	0.83	0.57	0.83	0.53	0.33	0.95	0.81	0.85	0.75

*^1^Total number of SNPs used as predictors in the model. For the SVM model, the highest accuracy was obtained using five SNPs including Gm10-1232205, Gm10-2240113, Gm10-214458, Gm10-1586434, and Gm11-63293.*

*^2^Overall prediction accuracy was calculated according to Eq. 1.*

*^3^Matthews Correlation Coefficient (MCC) was calculated according to Eq. 5.*

*^4^Class accuracy was calculated according to Eq. 2.*

*^5^Precision was calculated according to Eq. 3.*

*^6^Specificity was calculated according to Eq. 4.*

*The bold rows are the combination of SNPs with the highest accuracy.*

In the RF model, the highest accuracy (0.80) was obtained using 21 SNPs as predictors, including *Gm10-1232205, Gm10-2240113, Gm10-214458, Gm11-63293, Gm10-1586434, Gm10-4670275, Gm10-3465857, Gm15-13014539, Gm19-44761515, Gm13-35032818, Gm06-9668798, Gm16-6423098, Gm12-4883 456, Gm18-57126096, Gm16-31397286, Gm03-1718435, Gm11 -1620921, Gm06-3608127, Gm02-3774471, Gm10-39937578, Gm14-48703687*, and *Gm11-16996443.* Like the SVM model, *Gm10-1232205*, *Gm10-2240113*, *Gm10-214458*, *Gm10-1586434*, and *Gm11-63293* were among the most significant SNPs and the RF model using these five SNPs yielded an overall accuracy of 0.78. A total gain in overall classification accuracy of 11% was observed with the addition of 20 SNPs to the model using only *Gm10-1232205* (0.80 and 0.72, respectively) (Table 2). Similar to the SVM model, the highest gain in prediction accuracy by the addition of SNPs was observed in the moderate class (0.50–0.60). All prediction accuracy metrics were improved in the model with 21 SNPs. In the resistant class, an increase of 3.5, 15.2, and 7.1% was observed in class accuracy, precision, and specificity, respectively. In the moderate class, a more pronounced increase was observed in class accuracy and precision (20.0 and 252.9%, respectively). Increments proportional to the resistant class were observed in the susceptible class, including a gain of 7.5, 4.8, and 7.0% in class accuracy, precision, and specificity, respectively (Table 2).

**TABLE 2:** Summary of RF classification accuracy metrics based on the number of predictors.

# SNPs^1^	Overall accuracy^2^	MCC^3^	Tolerant			Moderate			Susceptible		
			Accuracy^4^	Precision^5^	Specificity^6^	Accuracy	Precision	Specificity	Accuracy	Precision	Specificity
2	0.73	0.50	0.85	0.59	0.84	0.50	0.17	0.96	0.79	0.83	0.71
3	0.74	0.54	0.85	0.59	0.84	0.51	0.33	0.98	0.80	0.83	0.69
4	0.77	0.58	0.85	0.59	0.84	0.59	0.63	0.98	0.84	0.87	0.78
5	0.78	0.59	0.86	0.62	0.86	0.57	0.57	0.98	0.84	0.86	0.76
6	0.77	0.57	0.86	0.62	0.86	0.54	0.43	0.97	0.84	0.86	0.76
7	0.77	0.58	0.86	0.62	0.86	0.54	0.43	0.97	0.84	0.86	0.76
8	0.76	0.59	0.83	0.62	0.87	0.56	0.40	0.95	0.84	0.86	0.76
9	0.77	0.58	0.86	0.61	0.85	0.58	0.56	0.97	0.84	0.87	0.78
10	0.76	0.57	0.84	0.60	0.85	0.60	0.50	0.95	0.84	0.88	0.80
11	0.79	0.60	0.86	0.62	0.86	0.60	0.60	0.97	0.86	0.88	0.80
12	0.79	0.62	0.87	0.63	0.87	0.62	0.64	0.97	0.86	0.88	0.80
13	0.79	0.61	0.87	0.63	0.87	0.62	0.64	0.97	0.86	0.88	0.80
14	0.79	0.63	0.86	0.62	0.86	0.62	0.64	0.97	0.85	0.88	0.80
15	0.78	0.60	0.86	0.62	0.86	0.59	0.63	0.98	0.84	0.86	0.76
16	0.78	0.56	0.87	0.63	0.87	0.58	0.56	0.97	0.84	0.86	0.76
17	0.79	0.55	0.85	0.67	0.90	0.61	0.67	0.98	0.82	0.84	0.71
18	0.79	0.53	0.84	0.68	0.90	0.61	0.67	0.98	0.82	0.83	0.69
19	0.79	0.59	0.82	0.67	0.90	0.60	0.55	0.96	0.84	0.85	0.73
20	0.79	0.56	0.84	0.68	0.90	0.60	0.55	0.96	0.85	0.86	0.75
**21**	**0.80**	**0.65**	**0.88**	**0.68**	**0.90**	**0.60**	**0.60**	**0.97**	**0.85**	**0.87**	**0.76**
22	0.79	0.57	0.88	0.67	0.89	0.60	0.60	0.97	0.84	0.86	0.76
23	0.79	0.57	0.86	0.66	0.89	0.58	0.56	0.97	0.84	0.86	0.75
24	0.78	0.60	0.86	0.66	0.89	0.59	0.63	0.98	0.82	0.84	0.71
25	0.78	0.59	0.87	0.65	0.88	0.56	0.44	0.96	0.84	0.86	0.76
26	0.77	0.57	0.85	0.63	0.87	0.60	0.55	0.96	0.83	0.86	0.76
27	0.79	0.59	0.86	0.68	0.90	0.60	0.60	0.97	0.82	0.85	0.73
28	0.78	0.60	0.84	0.65	0.89	0.61	0.67	0.98	0.82	0.84	0.71
29	0.78	0.59	0.84	0.65	0.89	0.59	0.63	0.98	0.82	0.84	0.71

*^1^Total number of SNPs used as predictors in the model. For the RF model, the highest accuracy was obtained using 21 SNPs including Gm10-1232205, Gm10-2240113, Gm10-214458, Gm11-63293, Gm10-1586434, Gm10-4670275, Gm10-3465857, Gm15-13014539, Gm19-44761515, Gm13-35032818, Gm06-9668798, Gm16-6423098, Gm12-4883456, Gm18-57126096, Gm16-31397286, Gm03-1718435, Gm11-1620921, Gm06-3608127, Gm02-3774471, Gm10-39937578, Gm14-48703687, and Gm11-16996443.*

*^2^Overall prediction accuracy was calculated according to Eq. 1.*

*^3^Matthews Correlation Coefficient (MCC) was calculated according to Eq. 5.*

*^4^Class accuracy was calculated according to Eq. 2.*

*^5^Precision was calculated according to Eq. 3.*

*^6^Specificity was calculated according to Eq. 4.*

*The bold rows are the combination of SNPs with the highest accuracy.*

**FIGURE 2:**
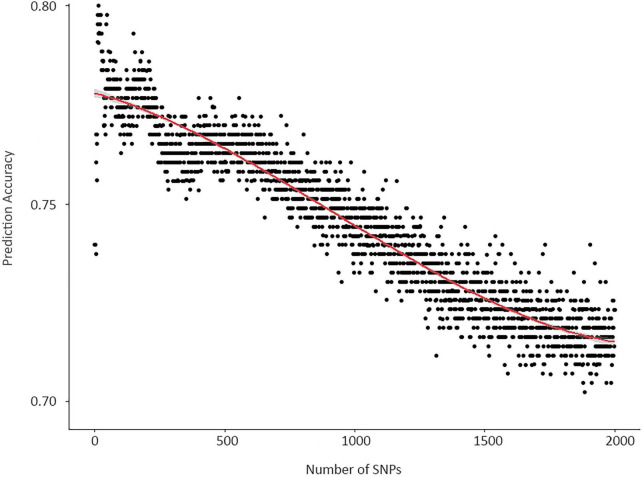
Prediction accuracy of RF models by the number of SNPs included as predictors.

Although RF is well-known for sustaining predictive performance under high dimensional data with multicollinearity, excessive noise among predictors, and unbalance between the number of predictors and the number of samples (Ishwaran et al., 2010; Chen and Ishwaran, 2012), a substantial decrease in overall accuracy by the addition of predictors was observed (Figure 2). Like the SVM model, the decrease in prediction accuracy is most likely due to the overfitting of the training set and poor reproducibility in the testing set. Due to computational limitations, the analysis included combinations of up to 2,000 SNPs instead of the entire set of 4,974 SNPs and was not performed for SVM.

#### Linear Model-Based Genome-Wide Association Studies

The SNPs *Gm10-1232205* and *Gm10-1586434* were detected in BLINK, FarmCPU, and ECMLM, as well as in SVM and RF (Table 3). In addition to these two SNPs located in genomic regions previously reported in the literature, *Gm10-2240113* was detected in the ECMLM, SVM, and RF and represents a potential additional marker-trait association in Chr. 10. The linear model-based GWAS methodologies did not detect *Gm10-214458* and *Gm11-63293* (Table 3). As shown in the previous section, these two SNPs contributed to increasing overall prediction accuracy when included in both SVM and RF models, and may represent additional marker-trait associations in Chrs. 10 and 11. These results show that BLINK, FarmCPU, and ECMLM perform well in detecting major effect SNPs, but lag in identifying minor effect alleles contributing to the observed phenotype. Both BLINK and FarmCPU models were able to adjust significance based on the presence of multicollinearity among SNPs, whereas the ECMLM model identified many correlated SNPs as significant associations which can lead to false-positive associations and an overall decrease in the predictive accuracy of the model.

**TABLE 3:** Summary of significant SNP-trait associations identified by GWAS using the BLINK, FarmCPU, and ECMLM models.

SNP	Chr^1^	Position^2^	MAF^3^	BLINK *p*-value^4^	FarmCPU *p*-value	ECMLM *p*-value	Significant models
*Gm10-1232205*	10	1,232,205	0.41	**<0.00000**	**<0.00000**	**<0.00000**	BLINK, FarmCPU, ECMLM
*Gm10-1586434*	10	1,586,434	0.20	**<0.00000**	**<0.00000**	**<0.00000**	BLINK, FarmCPU, ECMLM
*Gm10-1623075*	10	1,623,075	0.41	**<0.00000**	**<0.00000**	**<0.00000**	BLINK, FarmCPU, ECMLM
*Gm10-1426801*	10	1,426,801	0.37	**0.00006**	1.00000	**<0.00000**	BLINK, ECMLM
*Gm10-1475647*	10	1,475,647	0.14	**<0.00000**	1.00000	**<0.00000**	BLINK, ECMLM
*Gm14-3470438*	14	3,470,438	0.39	**0.00069**	1.00000	1.00000	BLINK
*Gm10-39827303*	10	39,827,303	0.49	0.22398	**0.00084**	1.00000	FarmCPU
*Gm10-1268065*	10	1,268,065	0.35	0.14341	1.00000	**<0.00000**	ECMLM
*Gm10-981062*	10	981,062	0.44	1.00000	1.00000	**<0.00000**	ECMLM
*Gm10-1341309*	10	1,341,309	0.25	1.00000	1.00000	**<0.00000**	ECMLM
*Gm10-925972*	10	925,972	0.47	1.00000	1.00000	**<0.00000**	ECMLM
*Gm10-2714130*	10	2,714,130	0.46	0.08015	1.00000	**<0.00000**	ECMLM
*Gm10-831916*	10	831,916	0.47	1.00000	1.00000	**<0.00000**	ECMLM
*Gm10-754804*	10	754,804	0.47	1.00000	1.00000	**<0.00000**	ECMLM
*Gm10-1051336*	10	1,051,336	0.37	1.00000	1.00000	**<0.00000**	ECMLM
*Gm10-14714*	10	14,714	0.11	0.90685	1.00000	**0.00042**	ECMLM
*Gm10-406427*	10	406,427	0.14	1.00000	1.00000	**0.00184**	ECMLM
*Gm10-2240113*	10	2,240,113	0.38	0.90685	1.00000	**0.00447**	ECMLM
*Gm10-2482570*	10	2,482,570	0.38	0.90685	1.00000	**0.01196**	ECMLM
*Gm10-2437001*	10	2,437,001	0.18	1.00000	1.00000	**0.02936**	ECMLM

*^1^Chromosome where the SNP is located.*

*^2^Position in the genome reported as basepairs.*

*^3^Minor allele frequency.*

*^4^False discovery rate (FDR)-adjusted p-values of each model to reduce false-positive associations.*

*Associations with a p-value lower than 0.05 are in bold.*

## Discussion

From a data analytics perspective, a GWAS is the identification of significant features controlling a respective trait of interest. Among thousands – often hundreds of thousands – of molecular markers distributed across the genome, the goal of the analysis is to select the most informative features and eliminate potential false-positive associations, a common drawback in high dimensional genomic data that presents multicollinearity, excessive noise among predictors, and unbalance between the number of predictors and the number of samples (Ishwaran et al., 2010; Chen and Ishwaran, 2012). Traditional GWAS models in plants are often vulnerable to overfitting, which leads to the detection of false-positive associations between molecular markers and the observed phenotype (Hayes, 2013; Korte and Farlow, 2013; Chen Z. et al., 2021). Overfitting happens when the model does not generalize well from observed to unseen data. This is caused by the model excessively capturing unintentional noise on the training set due to the presence of redundant predictors, and consequently yielding poor reproducibility on the testing set (Austin and Steyerberg, 2015; Ying, 2019). Feature selection is the process of identifying important predictors from the original variable set. This process is critical to avoid overfitting, improve model performance, and provide faster and more cost-effective models (Akarachantachote et al., 2014).

In this study, a novel GWAS pipeline that selects features based on the VIP followed by the elimination of highly correlated features and prediction accuracy in ML algorithms is proposed. The results indicated that major effect SNPs can be identified by the proposed methodology as well as the BLINK, FarmCPU, and ECMLM models. However, minor effect SNPs which improved the prediction accuracy of the two ML models were not detected in BLINK, FarmCPU, and ECMLM. In addition, a pronounced decrease in prediction accuracy was observed in the SVM model with the increment of SNPs as predictors, reaching the highest prediction accuracy in the model with five SNPs. RF, on the other hand, showed to be less vulnerable to overfitting and reached the highest prediction accuracy in the model with 21 SNPs. The ability of RF to include all markers, including low effect, highly correlated, and interacting markers to contribute to the model fit may explain the slight superior predictive accuracy by including more features in the model (Breiman, 2001; Díaz-Uriarte and Alvarez de Andrés, 2006; Pang et al., 2006; Ogutu et al., 2011). However, when the number of predictors exceeded 21, RF showed a steady decrease in prediction accuracy. This observation is important to guide future applications of genomic prediction, particularly for categorical phenotypes. As demonstrated in this research, identifying fewer but important predictors yielded higher prediction accuracy as compared to fitting the model with the highest number of predictors available. Yoosefzadeh-Najafabadi et al. (2021b) performed SVM, RF, ECMLM, and FarmCPU-based GWAS for soybean yield and its components including the number of reproductive nodes, non-reproductive nodes, total nodes, and total pods per plant. They found SVM to outperform all the other methodologies. However, as described by the authors, both RF and SVM results were based on variable importance and not on the prediction accuracy of each combination of SNPs. There are multiple reports of genomics and proteomics studies based on ML models that consider RF and SVM comparably good, and often superior to other ML models (Svetnik et al., 2003; Pang et al., 2006; Qi et al., 2006). The superiority of each algorithm is most likely based on the architecture of the dataset under study and investigating multiple algorithms should be encouraged to determine which is the most appropriate for a specific application.

Across SVM, RF, BLINK, FarmCPU, and ECMLM, the SNP *Gm10-1232205* was the most significant predictor associated with resistance to SRKN. It is located in a genomic region on Chr. 10 (1,232,205 bp) previously reported in the literature to be significantly associated with resistance to SRKN (Tamulonis et al., 1997; Li et al., 2001; Fourie et al., 2008). Tamulonis et al. (1997) found this QTL to explain 31% of the phenotypic variance, whereas Li et al. (2001) accounted this QTL for more than 55% of the phenotypic variance. In both studies, the resistance was assessed against SRKN race 3, and the source of resistance was PI 96354. Fourie et al. (2008), on the other hand, identified this QTL using SRKN race 2, a predominant race in soybean production areas of South Africa and accounted for more than 31% of the phenotypic variance. Within 50 kb of *Gm10-1232205*, two genes namely *Glyma.10g013700* (Universal Stress Protein) and *Glyma.10g013900* (Carbohydrate Metabolic Process) were identified as possible candidate genes associated with SRKN resistance. Universal Stress Proteins (USP) are involved in multiple cellular responses to biotic and abiotic stressors, ranging from ion scavenging, hypoxia responses, cellular mobility, and regulation of cell growth and development (Chi et al., 2019). *Glyma.10g013700* has been associated with the *Arabidopsis thaliana AT3G01520*, an adenine nucleotide alpha hydrolases-like superfamily protein that is involved in N-terminal protein myristoylation (Kim et al., 2015). The attachment of a myristoyl group enhances specific protein–protein interactions, thus playing an essential role in membrane targeting and signal transduction in plant responses to biotic and abiotic stressors (Podell and Gribskov, 2004; Traverso et al., 2008; Udenwobele et al., 2017). *Glyma.10g013900* has been associated with carbohydrate metabolic process with complete expression patterns in the root zone (Libault et al., 2010; Severin et al., 2010). It encodes a protein similar to β-xylosidase and is a member of the glycosyl hydrolase family, acting in the cell wall polysaccharide metabolism. Additional functions of glycosyl hydrolases are mobilization of energy, defense to biotic stressors, symbiosis, signaling, secondary plant metabolism, and metabolism of glycolipids (Minic, 2008). Gene expression analyses of soybean roots in response to SRKN infection have identified glycosyl hydrolase proteins to be overexpressed and likely associated with soybean’s ability to control the infection (Ibrahim et al., 2011; Beneventi et al., 2013). *Gm10-1586434* was also detected by SVM, RF, BLINK, FarmCPU, and ECMLM. This genomic region on Chr. 10 (1,586,434 bp) overlaps with reports from Tamulonis et al. (1997) and Li et al. (2001), as well as two more recent studies using bi-parental populations derived from PI 96354 (Pham et al., 2013) and PI 438489B (Xu et al., 2013). Pham et al. (2013) estimated this QTL to account for 50% of the phenotypic variance. Three cell wall modification candidate genes encoding for pectinesterase and extensin proteins were proposed, including *Glyma10g02090*, *Glyma10g02100*, and *Glyma10g02140* (Pham et al., 2013). Xu et al. (2013) pinpointed two candidate genes within this genomic region accounting for 23.6% of the phenotypic variance. They were *Glyma10g02150* and *Glyma10g02160* and encode a pectin methylesterase inhibitor (PMEI) and PMEI-pectin methylesterase, respectively (Xu et al., 2013).

In addition to this major QTL on Chr. 10 (1,018,664 to 1,881,027 bp) that has been well reported on the literature (Tamulonis et al., 1997; Li et al., 2001; Fourie et al., 2008; Pham et al., 2013; Xu et al., 2013; Passianotto et al., 2017), two new genomic regions on Chr. 10 associated with SRKN have been identified. *Gm10-2240113* is located at 2,240,113 bp and *Gm10-214458* is located at 214,458 bp of Chr. 10. These SNPs have been shown to increase both SVM and RF models’ prediction accuracy when included as a predictor, and may potentially represent additional minor effect marker-trait associations on Chr. 10. *Gm11-63293* is located at 63,293 bp of Chr. 11 and was found to increase the prediction accuracy of both SVM and RF models, however, it was not identified by either BLINK, FarmCPU, and ECMLM. This is the first time this genomic region has been reported to be associated with SRKN resistance. Within 200 bp of this SNP is located the gene *Glyma.11g001200*. Further investigation on Soybase.org (Grant et al., 2010) revealed this gene to be a leucine-rich repeat (LRR) family protein, a characteristic family protein that is required for plant resistance against viruses, bacteria, fungi, and nematodes. Interestingly, this family protein is similar to the *Mi* gene in tomato conferring resistance to SRKN (Milligan et al., 1998; Hwang and Williamson, 2003). Studies have identified the role of LRR-mediated intramolecular interactions in both nematode recognition and cell death signaling by the *Mi* gene (Milligan et al., 1998; Hwang and Williamson, 2003). Although the reported candidate genes are located nearby SNPs associated with the resistance of soybean to SRKN and show functions that make biological sense in the resistance pathway, additional studies involving gene function and analysis of the impact on the galling response should be conducted to validate this hypothesis.

## Conclusion

Although the major QTL on Chr. 10 can explain most of the phenotypic variance associated with SRKN resistance in soybean, additional minor effect marker-trait associations on Chrs. 10 and 11 were identified to improve the prediction accuracy of both SVM and RF models. The addition of minor effect SNPs enhanced the models’ predictive accuracy in classifying genotype response to SRKN, which could improve the ability of plant breeding programs to identify resistant genotypes through marker-assisted selection and/or genomic prediction early in the breeding pipeline. Interestingly, a decrease in classification accuracy was observed for the ML models as the number of SNPs included in the analysis increased, which reinforces the importance of limiting the unbalance between the number of predictors and the number of samples resulting in overfitting and poor reproducibility of the results. Minimal diversity and evolution are expected since SRKN are parthenogenic nematodes. However, resistance breakdown has been observed in tomatoes against the *Mi* gene (Eddaoudi et al., 1997). Resistance-breaking population in soybean could dramatically impact the soybean value chain because of the degree of yield losses caused by SRKN as well as the lack of alternative management options (Vieira et al., 2021). Expanding the basis of the genetic resistance to SRKN can potentially reduce the selection pressure over the major QTL on Chr. 10, and as demonstrated in this study, result in higher levels of resistance.

## Data Availability Statement

The raw data supporting the conclusions of this article will be made available by the authors, without undue reservation.

## Author Contributions

PC and GS contributed to conception, design, and funding resources of the study. AH and ZL contributed to the phenotyping of soybean lines used in this study. CC, MU, TV, DL, and HN contributed to the genotyping of the soybean lines used in this study. CC, JZ, and JFZ contributed to the statistical analysis of this study. CC and JZ wrote the first draft of the manuscript. All authors contributed to manuscript revision, read, and approved the submitted version.

## Conflict of Interest

The authors declare that the research was conducted in the absence of any commercial or financial relationships that could be construed as a potential conflict of interest.

## Publisher’s Note

All claims expressed in this article are solely those of the authors and do not necessarily represent those of their affiliated organizations, or those of the publisher, the editors and the reviewers. Any product that may be evaluated in this article, or claim that may be made by its manufacturer, is not guaranteed or endorsed by the publisher.
